# In situ metalation of free base phthalocyanine covalently bonded to silicon surfaces

**DOI:** 10.3762/bjnano.5.231

**Published:** 2014-11-25

**Authors:** Fabio Lupo, Cristina Tudisco, Federico Bertani, Enrico Dalcanale, Guglielmo G Condorelli

**Affiliations:** 1 Dipartimento di Scienze Chimiche, Università di Catania and INSTM UdR di Catania, v.le A. Doria 6, 95125 Catania, Italy; 2 Dipartimento di Chimica and INSTM UdR di Parma Università of Parma Parco Area delle Scienze 17/A, 43124 Parma, Italy

**Keywords:** metalation, phthalocyanine, silicon surface, surface functionalization, X-ray photoelectron spectroscopy (XPS)

## Abstract

Free 4-undecenoxyphthalocyanine molecules were covalently bonded to Si(100) and porous silicon through thermic hydrosilylation of the terminal double bonds of the undecenyl chains. The success of the anchoring strategy on both surfaces was demonstrated by the combination of X-ray photoelectron spectroscopy with control experiments performed adopting the commercially available 2,3,9,10,16,17,23,24-octakis(octyloxy)-29*H*,31*H*-phthalocyanine, which is not suited for silicon anchoring. Moreover, the study of the shape of the XPS N 1s band gave relevant information on the interactions occurring between the anchored molecules and the substrates. The spectra suggest that the phthalocyanine ring interacts significantly with the flat Si surface, whilst ring–surface interactions are less relevant on porous Si. The surface-bonded molecules were then metalated in situ with Co by using wet chemistry. The efficiency of the metalation process was evaluated by XPS measurements and, in particular, on porous silicon, the complexation of cobalt was confirmed by the disappearance in the FTIR spectra of the band at 3290 cm^−1^ due to –NH stretches. Finally, XPS results revealed that the different surface–phthalocyanine interactions observed for flat and porous substrates affect the efficiency of the in situ metalation process.

## Introduction

Free (Pc) and metallophthalocyanines (M–Pc) are molecules of great interest because of their versatile optical and electronic properties as well as their thermal stability [1]. These properties make them attractive molecular materials for applications in photovoltaic cells [2], sensing devices [3–4], catalysis [5], cancer therapy [6] and molecular electronics [3,7–8]. The most promising architecture for the exploitation of the potentialities of Pc and M–Pc is the organization of the molecules in a suitable and accessible way on a solid surface. Therefore, phthalocyanine thin films have been deposited by using different techniques including Langmuir–Blodgett deposition [9], spin-coating [10] and vapor deposition [10–11]. Well-organized monolayers and multilayers have been also obtained through self-assembly [12–13]. Among the various approaches adopted to organize phthalocyanines on surfaces, covalent grafting on H-terminated silicon through hydrosilylation reaction has the advantage to form robust and highly stable Si–C bonds. For this reason, a device based on silicon-grafted molecules possesses a much greater robustness and reliability compared to van der Waals films or Au-bonded layers, which makes these systems suited for application in aggressive environments [14–16]. In addition, the possible use of differently doped silicon substrates could influence the electronic properties of grafted Pc and M–Pc [3], and, in turn, the device properties. Furthermore, the overall chemical and physical properties of M–Pc can be easily tuned by varying the nature of the coordinated metal, thus making phthalcyanine-based systems suitable for a wide range of applications. In particular, transition metal Pc have attracted great interest for their optical and magnetic properties [8,17] as well as for their potential catalytic [5] and sensing applications [4]. Various metallophthalocyanines (Zn, Fe, Co, Cu, Sn) have been deposited as monolayers and multilayers on various surfaces [13,18] and, in some cases, free base Pc have been metalated directly on the metal surface from vapor-deposited atoms [19–20]. However, no report of the direct metalation of covalently bonded Pc on inorganic surfaces has been reported, yet.

**Figure 1:**
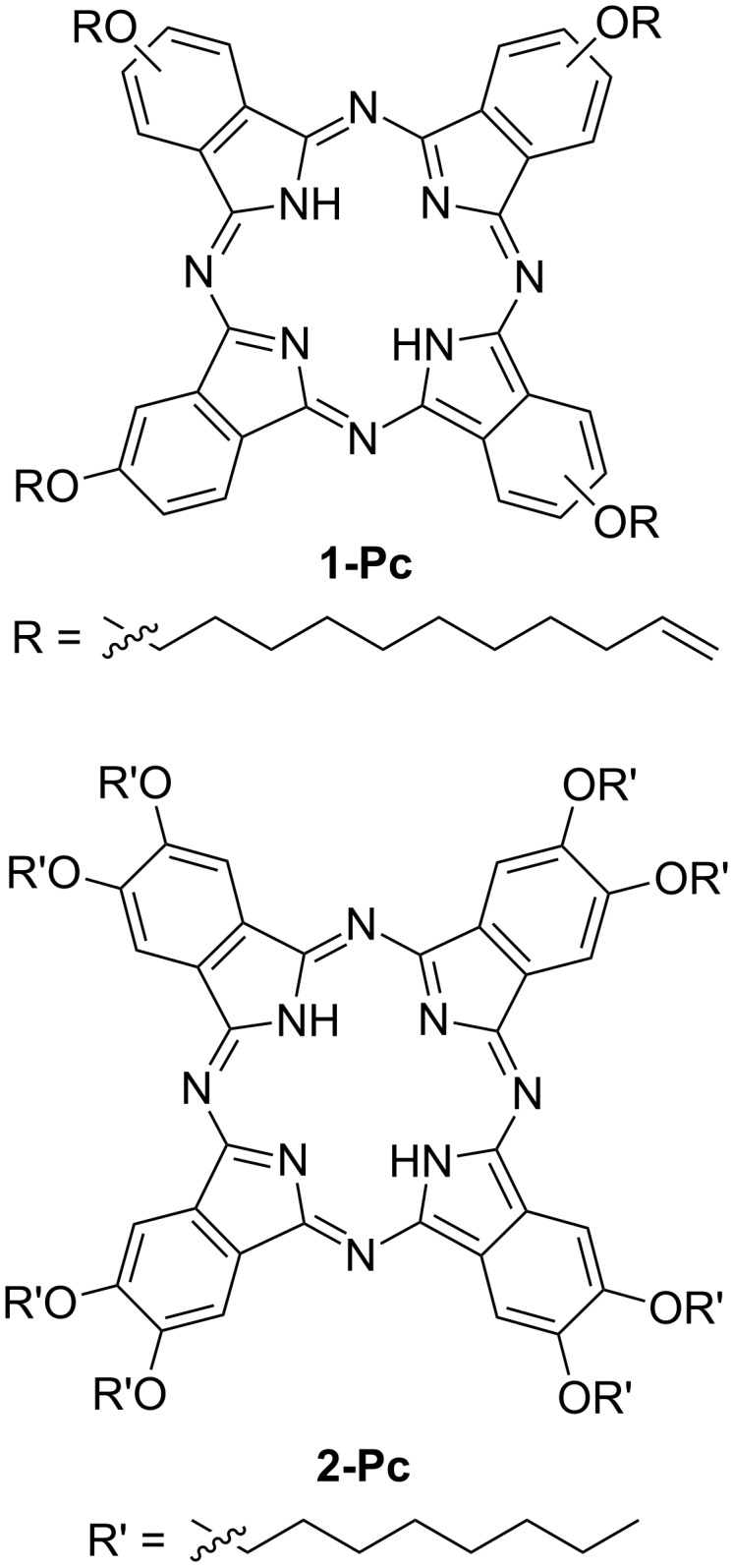
Chemical structures of **1-Pc** and **2-Pc**.

In this work we study the silicon grafting of the tetra-4-(ω-undecenyloxy)phthalocyanine (thereafter **1-Pc**) (Figure 1) and its interaction with a silicon surface. **1-Pc** was synthesized to allow for a silicon grafting by functionalization with four undecenyl chains each having a terminal double bond. Phthalocyanine covalent anchoring was performed through thermic hydrosilylation on flat Si(100) and on porous silicon (**Si-1-Pc** and **PSi-1-Pc**, respectively). The success of the anchoring strategy on both surfaces was demonstrated by the combination of X-ray photoelectron spectroscopy (XPS) with control experiments performed adopting the commercially available 2,3,9,10,16,17,23,24-octakis(octyloxy)-29*H*,31*H*-phthalocyanine (thereafter **2-Pc**), which is not suited for silicon anchoring (Figure 1).

**1-Pc** covalently bonded to silicon surface was in situ metalated with Co by using a solution of cobalt chloride. The direct formation of Co-Pc on flat and porous Si **(Si-Co-Pc** and **PSi-Co-Pc**, respectively) was monitored by XPS and FTIR. In particular, for phthalocyanines anchored on porous Si, transmission FTIR represents a suitable technique to monitor the formation M–Pc through the disappearance of the band at 3290 cm^−1^, corresponding to the pyrrolic –NH streches [21]. Differences in the metalation efficiency between porous and flat silicon were evaluated by XPS and explained in terms of different surface interactions.

## Results and Discussion

### Synthesis of **1-Pc**

Phthalocyanine **1-Pc** was prepared according to a slightly modified literature procedure [22] starting from the 4-(ω-undecenyloxy)phthalonitrile in refluxing 1-pentanol in presence of a catalytic amount of 1,8- diazabicyclo[5.4.0]undec-7-ene (DBU) as a basic catalyst. The target compound was isolated in 58% yield as a dark-green powder after purification. **1-Pc** was successfully characterized by ^1^H NMR and MALDI–TOF mass spectrometry (see Experimental section).

#### XPS characterization of Si-bonded phthalocyanine

Covalent anchoring of **1-Pc** on flat Si(100) and porous Si was performed through thermally activated hydrosilylation and the functionalized samples (**Si-1-Pc** and **PSi-1-Pc**, respectively) were characterized through XPS. In addition, further experiments were performed to demonstrate that the surface anchoring is not due to physisorption but it is due to the hydrosilylation reaction. Control samples were, therefore, prepared by treating flat and porous silicon surfaces with a phthalocyanine (**2-Pc**), in which no double bonds are present in the lateral chains, under the same experimental conditions adopted for **1-Pc** anchoring. Elemental compositions of **1-Pc** and **2-Pc** treated samples are reported in Table 1.

**Table 1:** Atomic compositions (%) evaluated through XPS of **1-Pc** treated flat (**Si-1-Pc**) and porous (**PSi-1-Pc**) silicon samples. Analogous samples obtained from **2-Pc** treatment (**Si-2-Pc** and **PSi-2-Pc**) have been also reported as control experiments.

	**Si-1-Pc**	**Si-2-Pc**	**PSi-1-Pc**	**PSi-2-Pc**

C	36.6	11.8	56.5	30.5
N	2.4	0.5	4.6	0.6
Si	38.8	46.7	29.6	34.3
O	22.2	41.0	9.3	34.6

XPS data show that **Pc**-related signals (C 1s and N 1s) are higher for the **1-Pc** treated samples compared to the **2-Pc** treated samples. Since the C 1s signal is affected by the presence of ubiquitous adventitious carbon [23–24], the success of Pc anchoring route can be evaluated from the N 1s signal, which is very low for **Si-2-Pc** and **PSi-2-Pc** samples while it is about 5 and 8 times higher for **1-Pc** treated surfaces. These data point to a surface-anchoring process determined by the hydrosilylation reaction of the double bonds while physisorption phenomena play a much less relevant role.

The surface density of **1-Pc** on flat Si(100) was estimated from XPS data (Table 1) [25–27]. The obtained value, ca. 2 × 10^13^ molecules/cm^2^, points to a molecular footprint of 5 nm^2^ for each molecular unit, which is intermediate between the cross-sectional areas expected for a configuration with the side chains vertical with respect to the phthalocyanine ring (ca. 1 nm^2^) and a configuration in which all four alkyl side chains are full extended in the same plane of the ring (ca. 9 nm^2^).

**Figure 2:**
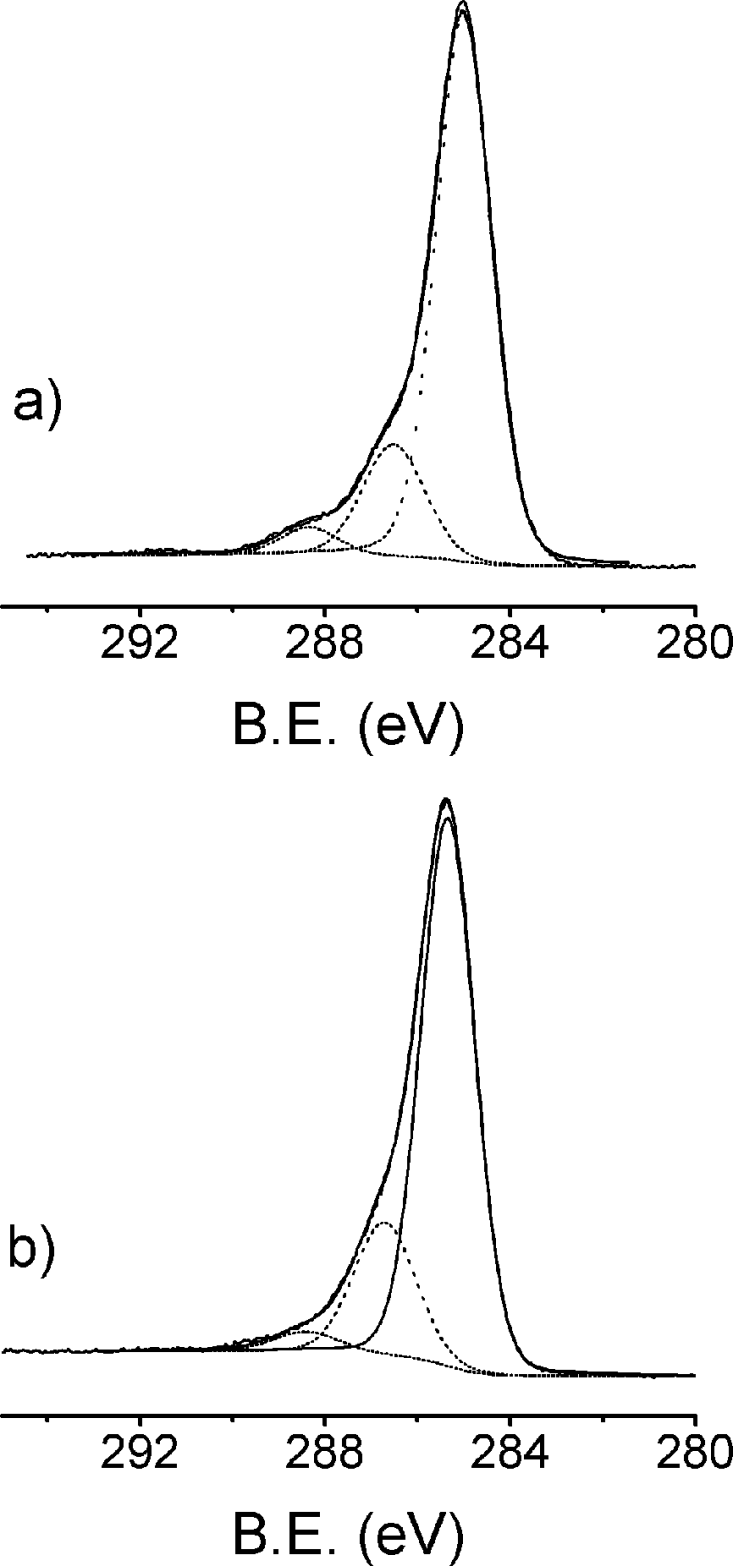
C1s XPS spectral region of **Si-1-Pc** (a) and **PSi-1-Pc** (b).

Useful information about the nature of the grafted layers was obtained from high-resolution spectra of the relevant photoemission bands. Figure 2 reports the C 1s photoelectron spectra of **Si-1-Pc** (a) and **PSi-1-Pc** (b). The observed bands do not show significant differences between flat and porous samples. For both samples, a careful deconvolution of the band envelope reveals three components: a main peak centered at 285.0 eV, due to both aliphatic and aromatic carbons [11]; a band at a binding energy (B.E.) of 286.5 eV due to the pyrrole carbons and to the shake-up related to benzene carbons, in tune with literature data [11]; and finally, a band at 288.3 eV (288.1 eV for **PSi-1-Pc**) due to the shake-up transition associated with the photoionization of pyrrole [11].

**Figure 3:**
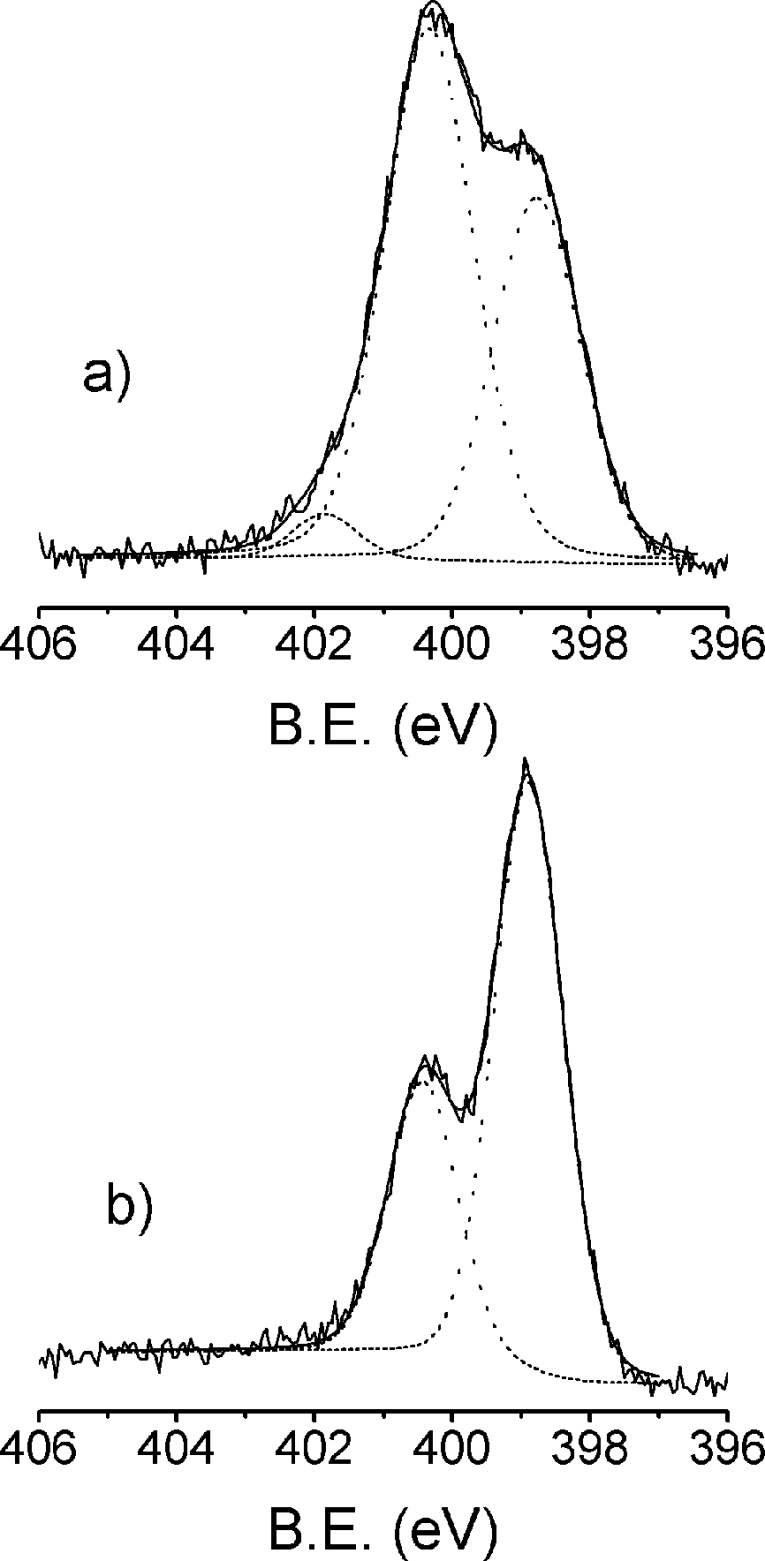
N 1s XPS spectral region of **Si-1-Pc** (a) and **PSi-1-Pc** (b).

The N 1s XPS spectral regions of **Si-1-Pc** and **PSi-1-Pc** are reported in Figure 3a and 3b, respectively. The spectrum collected from **Si-1-Pc** shows two bands, at B.E. values of 398.8 and 400.4 eV. The first signal is due to non-protonated pyrrolic nitrogen atoms and due to iminic bridges, whilst the high B.E. signal is due to protonated pyrrolic nitrogen atoms [28–30]. However the intensity ratio between the 398.8 and 400.6 eV bands is 2:3, which is significantly different from the value (3:1) expected for free-base phthalocyanines [28–30]. The increase of the high B.E signal can be explained as a consequence of the interaction with the silicon surface. The effects of various surfaces on the shape of the N 1s band have been already observed and discussed for other metal phthalocyanine monolayers adsorbed on oxide semiconductors [28–30] and, recently, reported also for double-decker complexes on silicon [31]. In general, according to the mentioned studies, the interaction between the fraction of anchored phthalocyanine lying down close to the surface and the semiconductors surface itself, induce an electron depletion in the phthalocyanine ring and, in turn, a high energy shift (about 1.5 eV) from 398.8 to about 400.3 eV of the main N 1s component due to deprotonated nitrogen atoms [28–29,31]. Possible local interactions (i.e., H-bonds) between the phthalocyanine ring lying down close to the surface and the Si surface itself could contribute to a similarly high B.E. shift [32] and cannot be excluded. In any case, the surface-induced shift can explain the increase of the component at 400.4 eV and also the presence of a low shoulder at around 401.8 eV due to protonated nitrogen atoms.

The N 1s XPS spectrum of **Psi-1-Pc** (Figure 3b) shows a different situation. The N 1s band consists of the same two components at 398.6 and 400.4 eV observed for **Si-1-Pc**, but in this case the component ratio is 2:1, which is closer to the value expected for free phthalocyanine, thus indicating that there are no strong interactions between the surface of PSi and the Pc molecules.

#### Metalation of SAM

**Figure 4:**
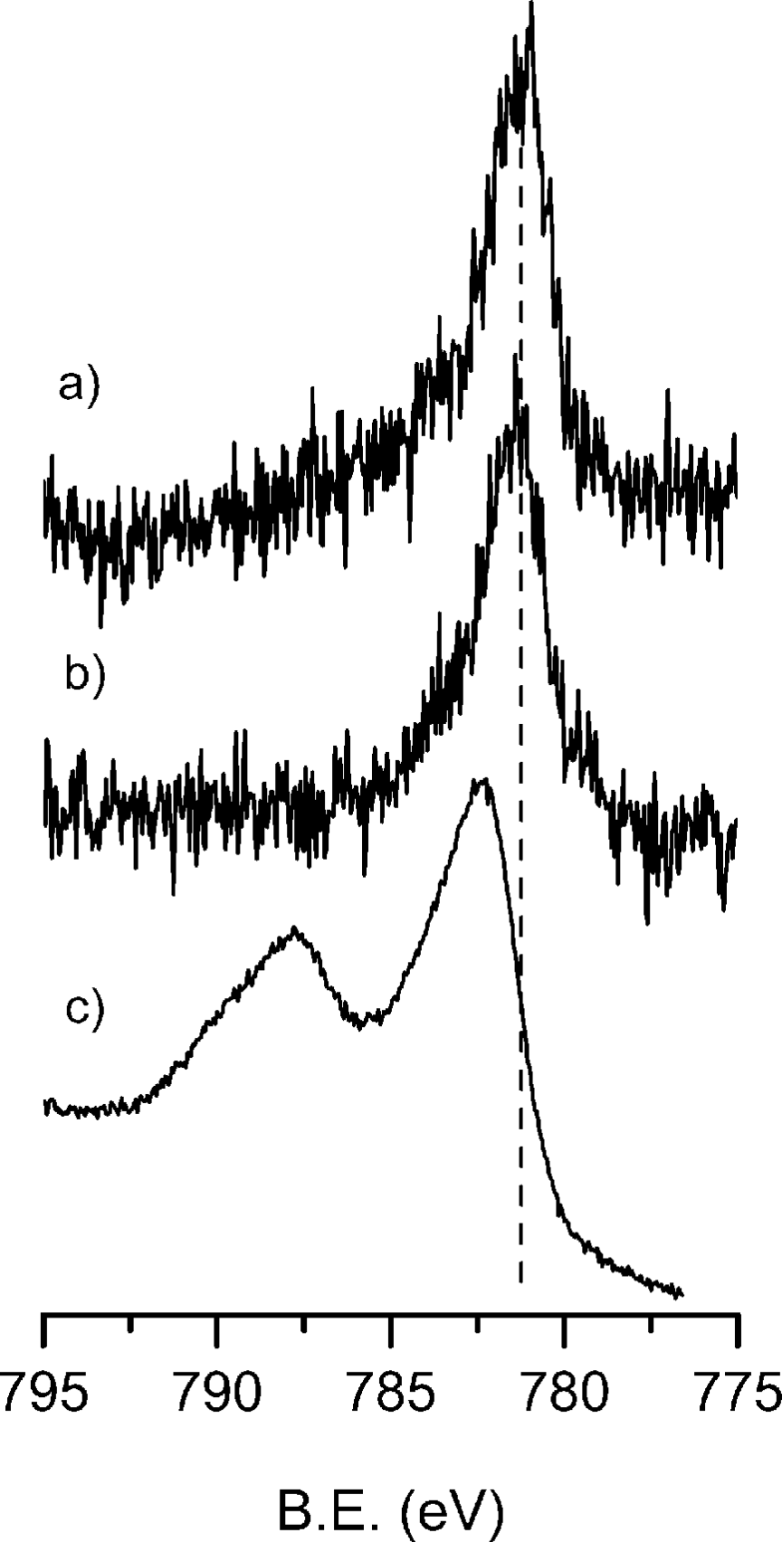
Co 2p3/2 XPS spectral region of **Si-Co-Pc** (a) and **PSi-Co-Pc** (b). The Co 2p3/2 region of CoCl_2_ powder (c) has been added as reference.

The possibility to induce a direct metalation of the grafted Pc was explored for both **Si-1-Pc** and **PSi-1-Pc** samples. **1-Pc** that was covalently bonded to Si and PSi surfaces has been treated with a solution of CoCl_2_ in diglyme in the presence of triethylamine and then accurately sonicated to remove any physisorbed salt. XPS characterization of cobalt treated **Si-1-Pc** and **PSi-1-Pc** samples (**Si-Co-Pc** and **PSi-Co-Pc**, respectively) clearly showed the presence of Co, whilst no Cl could be detected (Cl content < 0.1% noise level). Similar bands centered at 781.2 eV are present in the spectra of both **Si-Co-Pc** and **PSi-Co-Pc** (Figure 4a and Figure 4b). Although this band position is consistent with the presence of Co(II) atoms, the peak position and, in particular, the absence of the intense shake-up typical of Co(II) compounds such as CoCl_2_ (Figure 4c) indicate that Co signal is not due to physisorbed CoCl_2_. The observed band shape and position are consistent with spectra reported for Co-phthalocyanine thin films [33–35].

In addition, complexation efficiency was estimated from the N/Co atomic ratio determined through XPS. Considering a theoretical N/Co ratio of 8 expected for 100% of complexation, N/Co ratios obtained for **Si-Co-Pc** and **PSi-Co-Pc** (28.6 and 10.7, respectively) indicate a percentage of metalation of 28% and 75%, respectively.

**Figure 5:**
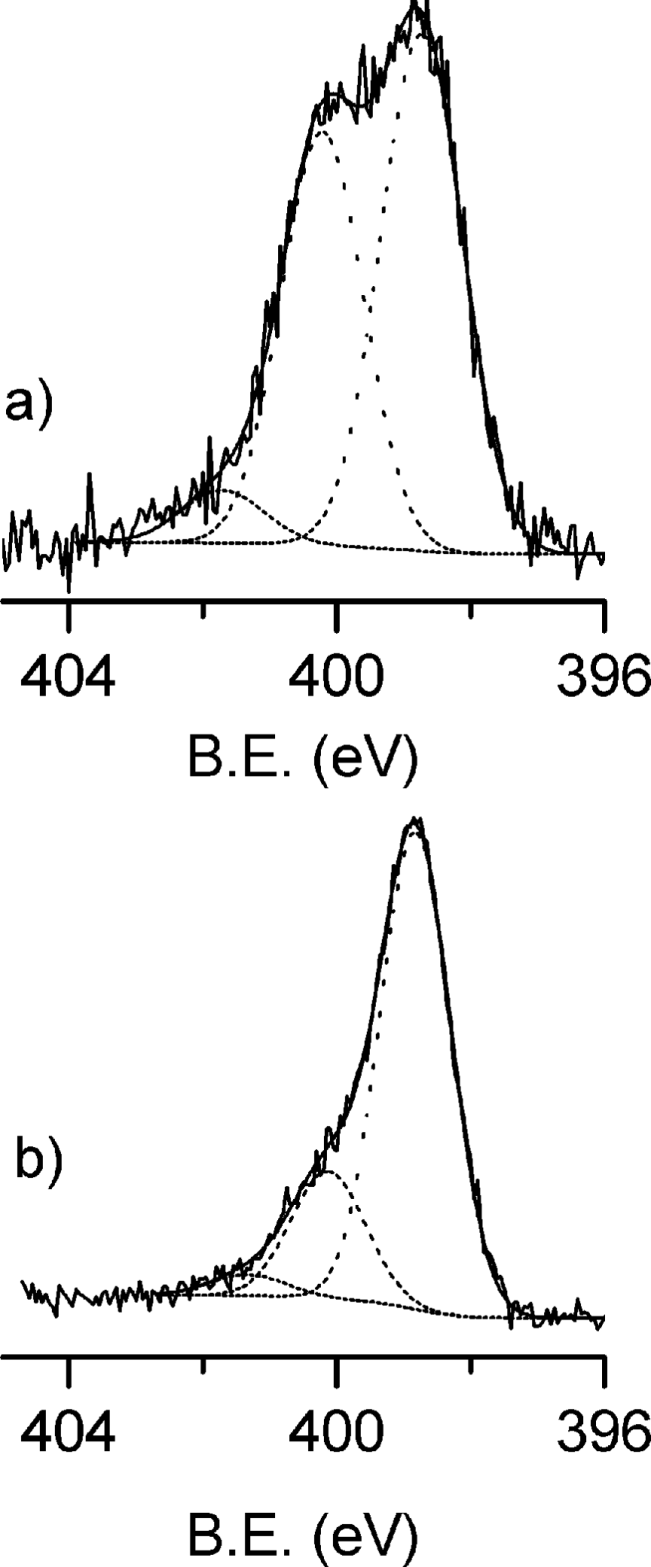
N 1s XPS region of **Si-Co-Pc** (a) and **PSi-Co-Pc** (b).

Further indication of Co complexation in the Pc-ring was obtained from the analysis of the N 1s spectra (Figure 5) after metalation. Clearly the presence of cobalt gives rise to a modification on the N 1s band shape compared to spectra before metalation (Figure 3). As expected, after metalation the intensity of the component at 400.4 eV due to –NH pyrrolic nitrogen atoms decreases compared to the low B.E. component at 398.8 eV since the metal coordination is associated to the deprotonation of pyrrolic nitrogen atoms to form N–Co [36]. In particular, for **PSi-Co-Pc,** for which the metalation efficiency is higher than that of **Si-Co-Pc,** the 400.4 eV signal becomes much lower and the spectrum becomes similar to the typical spectra of M–Pc in which a single band at low B.E. is present [36]. Note, in addition, that eventual interferences due to triethylamine physisorption on **PSi,** which would lead to the increase of the N 1s component around 400 eV**,** can be ruled out since the reverse trend was observed for **PSi-1-Pc**. Overall the metalation appears more efficient in the case of the porous silicon substrate compared to flat Si(100). This behavior is likely to be associated to the different surface interactions observed for **Si-1-Pc** and **PSi-1-Pc**. In the case of a flat substrate the proposed strong surface interaction of the fraction of **1-Pc** lying down close the substrate prevents an efficient insertion of Co in the Pc ring, whilst in the case of porous samples, less strong surface interactions allow for a more efficient metalation.

**Figure 6:**
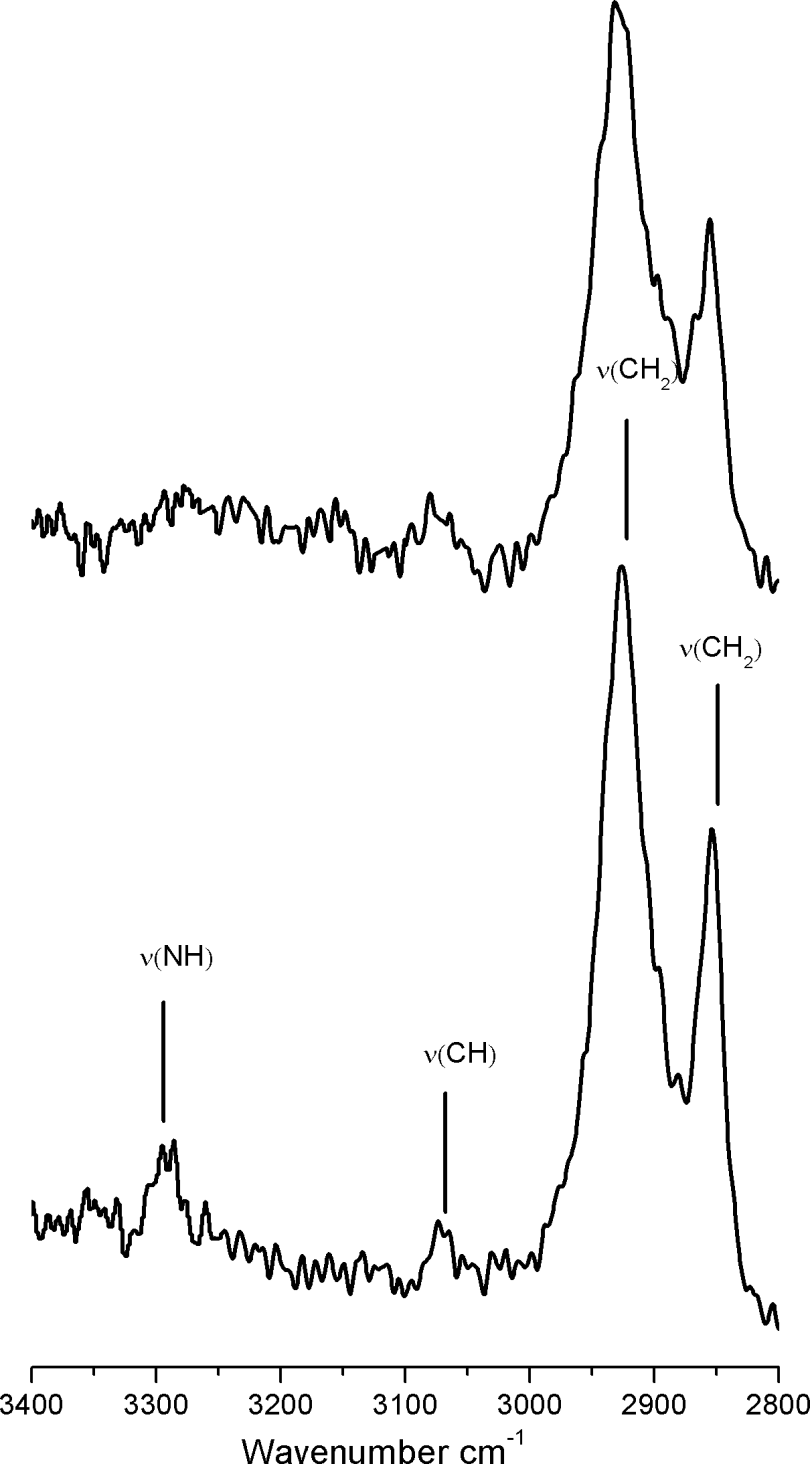
FTIR spectral region between 3400–2800 cm^−1^ (CH*_x_* stretching region) of **PSi-1-Pc** (below) and **PSi-Co-Pc** (above).

Further information about the grafting of **1-Pc** on porous silicon and about the in situ metalation could be obtained from transmission FTIR spectra by taking advantage of the high surface area of **PSi**. Figure 6 compares the FTIR region of 3400–2800 cm^−1^ in which –CH*_x_* and –NH stretches are present before and after the metalation. Typical bands present before the metalation are the strong CH_2_ stretches ν_as_(CH_2_) at 2925 cm^−1^ and ν_sym_(CH_2_) at 2854 cm^−1^, the weak =CH stretches ν(=CH) of the aromatic rings at 3070 cm^−1^ and the characteristic –NH stretch of pyrrolic nitrogen atoms at about 3290 cm^−1^. After metalation, –NH stretch vibrations cannot longer be clearly detected, whilst the other bands are still observed. Since triethylamine is unable to deprotonate the phthalocyanine, the absence of N–H bonds is exclusively due to the Co complexation [21].

## Conclusion

The results presented here report on a grafting route to covalent anchor phthalocyanine on flat and porous silicon surfaces. The grafting route was validated by XPS characterization and control experiments that were performed by adopting a phthalocyanine inert towards hydrosilylation. XPS results also suggest that on flat substrates a relevant fraction of phthalocyanine interacts significantly with the silicon surface, thus inducing evident modifications of the N 1s band shape. On porous silicon, surface interactions are less relevant and the N 1s band shape is similar to the typical shape of free Pc.

In situ complexation of Co was achieved with phthalocyanine bonded to both flat and porous silicon surfaces. However, the metalation efficiency is higher in the case of porous samples. These differences were attributed to the different surface interactions observed for the two samples. If strong surface interactions are present, as in the case of flat silicon, metalation is less efficient, whilst if Pc does not interact significantly with the surface, as in the case of PSi, the efficiency of the metalation improves. Although further work is required to better clarify the nature of interaction between the silicon surface and the molecular system, these results represent a step forward in the understanding of the chemistry of phthalocyanine covalently bonded to inorganic surfaces.

## Experimental

### Reagents

All chemicals, unless otherwise noted, were commercially available and used as received. Water used for porous silicon and monolayer preparations was a Milli-Q grade (18.2 MΩ·cm) with a final filtering step through a 0.22 μm filter. **2-Pc** was purchased from Aldrich chemicals.

#### **1-Pc** synthesis

To a stirred solution of 4-(ω-undecenyloxy)phthalonitrile [22] (300.0 mg, 1.01 mmol) in 1-pentanol (10 mL) a catalytic amount of DBU was added. The resulting solution was stirred at 135 °C for 14 h under N_2_. After cooling, methanol was added to the residue until a precipitate formed. The green finely dispersed mixture was filtered off and purified by flash chromatography (DCM as eluent) to give **1-Pc** as a green solid (174.0 mg, 58% yield). ^1^H NMR (400 MHz, CDCl_3_) δ 6.91–6.72 (br), 5.99–5.93 (m, 4H), 5.16–5.05 (m, 8H), 3.98 (bs, 8H), 2.22 (m, 8H), 2.01 (bs, 8H), 1.71–1.54 (m, 48H); MALDI–TOF (*m*/*z*): [M]^+^ calcd for C_76_H_98_N_8_O_4_, 1186.77; found, 1186.79.

#### Preparation of **Si-1-Pc** and **PSi-1-Pc**

The anchoring of **1-Pc** on a single crystalline, Czochralski grown, p-type boron-doped, (100)-oriented silicon substrate was performed through a well establish thermal hydrosilylation route [37–38]. Similarly to the procedure described in [38], Si(100) substrates were first cleaned with ‘‘piranha’’ solution (H_2_SO_4_ (30%)/H_2_O_2_ 70:30, v/v) at room temperature for 12 min, rinsed in double distilled water for 2 min, etched in 2% hydrofluoric acid for 90 s, washed with double distilled water for 20 s, accurately dried with pre-purified N_2_, and immediately placed in a three neck flask containing 10 mL of anhydrous mesitylene (Sigma-Aldrich) in which was dissolved 25 mg of **1-Pc** (2.1 mmol/L). The solution was then refluxed at 190 °C for 2 h, under slow N_2_ bubbling. After cooling to room temperature, the substrates were removed from the flask, rinsed, and repeatedly sonicated in dichloromethane, pentane, and toluene to remove any residual unreacted Pc.

Porous Si (PSi) was prepared by a metal-assisted chemical etching method [39]. A Czochralski grown, p-type boron-doped, Si(100) substrate was immersed in an aqueous solution of 0.14 M HF and 5 × 10^−4^ M in AgNO_3_ for 5 min, washed in water and then immersed for 1 min in a solution of HF (65%)/H_2_O_2_ (25%)/H_2_O (10%), washed in water and then left for 1 h in a solution of HF (20%)/H_2_O (80%). At the end, the substrate was washed, dried and placed in a three-neck flask containing a solution of **1-Pc** in mesitylene (2.1 mmol/L) and treated as described for flat Si(100) grafting. In this case the reaction time was increased to 4 h.

Control experiments were performed by placing non-etched Si(100) or PSi substrates in a three-neck flask containing a **2-Pc** solution (2.1 mmol/L) in mesitylene and treated as described for Si(100) or PSi grafting.

#### Direct metalation

Metalation of the silicon-anchored Pc was obtained by wet chemistry. The freshly prepared **Si-1-Pc** and **PSi-1-Pc** were immersed in a flask containing a cobalt solution and then refluxed at 160 °C for 8 h, under slow N_2_ bubbling. The solution was prepared by dissolving 80 mg of CoCl_2_ in 20 mL of anhydrous diglyme and 3 mL of TEA (triethylamine). The substrates were finally washed several times with diglyme and sonicated first in CH_2_Cl_2_ and then in EtOH.

#### Material characterisations

X-ray photoelectron spectra (XPS) were measured at a take-off angle of 45°, relative to the surface plane, with a PHI 5600 Multi Technique System (base pressure of the main chamber 2 × 10^−10^ Torr). The spectrometer is equipped with a dual Mg/Al standard X-ray source and a monochromatized Al source, a spherical capacitor analyzer (SCA) with a mean diameter of 279.4 mm. The samples were excited with monochromatized Al Kα radiation. The XPS peak intensities were obtained after Shirley background removal. No relevant charging effect was observed. Freshly prepared samples were quickly transferred to the XPS main chamber. The XPS binding energy scale was calibrated by centering the C 1s peak (due to hydrocarbon moieties and adventitious carbon) at 285.0 eV [24,40]

Infrared attenuated total reflectance spectra of the monolayers were recorded by using a Jasco FT/IR-430 spectrometer (100 scans collected per spectrum, scan range 560–4000 cm^−1^, resolution 4 cm^−1^).
